# Neutrophil Heterogeneity as Therapeutic Opportunity in Immune-Mediated Disease

**DOI:** 10.3389/fimmu.2019.00346

**Published:** 2019-03-04

**Authors:** Ricardo Grieshaber-Bouyer, Peter A. Nigrovic

**Affiliations:** ^1^Division of Rheumatology, Immunology and Allergy, Brigham and Women's Hospital, Boston, MA, United States; ^2^Division of Immunology, Boston Children's Hospital, Boston, MA, United States

**Keywords:** neutrophil, immune mediated disease, therapeutic opportunities, granulopoiesis, neutrophil migration, CD177, autoimmune, therapeutic targeting

## Abstract

Neutrophils are versatile innate effector cells essential for immune defense but also responsible for pathologic inflammation. This dual role complicates therapeutic targeting. However, neither neutrophils themselves nor the mechanisms they employ in different forms of immune responses are homogeneous, offering possibilities for selective intervention. Here we review heterogeneity within the neutrophil population as well as in the pathways mediating neutrophil recruitment to inflamed tissues with a view to outlining opportunities for therapeutic manipulation in inflammatory disease.

## Introduction

Circulating leukocytes have long been categorized by microscopic appearance as lymphocytes, monocytes, and granulocytes. In the late 1870s, Paul Ehrlich distinguished neutrophils from eosinophils and basophils using aniline stains (1). Neutrophils are diverse in phenotype, although the understanding of this heterogeneity remains relatively basic. Related challenges include the relative homogeneity of neutrophils on microscopic examination, a paucity of surface markers defining clear-cut subgroups, short *in vitro* lifespan, and susceptibility to activation with manipulation. Experiments using newer techniques such as mass cytometry and RNAseq often exclude neutrophils by restricting analysis to cryopreserved peripheral blood mononuclear cells (PBMC) or to cells with high mRNA content. The definition of subpopulations within neutrophils has thus lagged behind work in other lineages.

This gap does not reflect doubt about the immune importance of neutrophils. Quantitative and qualitative neutrophil defects expose patients to a high risk of infection, amply displayed in both congenital and acquired neutrophil disorders (2, 3). In mice, neutropenia resulting, for example, from congenital deficiency of the transcription factor *Gfi1* translates into high mortality from bacterial pathogens (4). Safety concerns translate into an understandable reluctance to target neutrophils therapeutically.

The failure to develop such strategies passes up potential opportunities to intervene in human disease. Neutrophils feature prominently in pathogenic sterile inflammation. For example, neutrophils are ubiquitous in the inflamed joint in rheumatoid arthritis (RA), in peritonitis associated with familial Mediterranean fever, and in the neutrophilic dermatoses (5–7). Among the pediatric rheumatic diseases, neutrophils are uniformly present in inflamed juvenile idiopathic arthritis (JIA) synovial fluid and have been implicated in the pathogenesis of the childhood-restricted vasculitis Kawasaki disease (8–11) While presence alone does not establish causation, evidence for a pathogenic role is frequently compelling. For example, experimental arthritis is abrogated in mice that lack neutrophils or with impaired neutrophil migration or function (12–15). Analogous studies implicate neutrophils as key effectors in a myriad of immune mediated diseases, including neuroinflammation, colitis, and bullous pemphigoid (16, 17). Neutrophils therefore remain an interesting drug target.

The therapeutic challenge is to develop strategies that preserve the defensive contribution of neutrophils while hindering their capacity to mediate sterile inflammation. Selectivity may be achieved by leveraging differences within the neutrophil population, in the way that cancer chemotherapy for targets cells that undergo frequent mitosis or bear specific mutations. Opportunities to drive a “wedge” between protective and pathogenic functions could also arise through differences in effector pathways that neutrophils engage in responding to sterile and septic triggers. This review will explore these possibilities with a view to highlighting potential treatment targets in neutrophils.

## Neutrophil Biology: Ontogeny and Lifecycle

**Figure 1:**
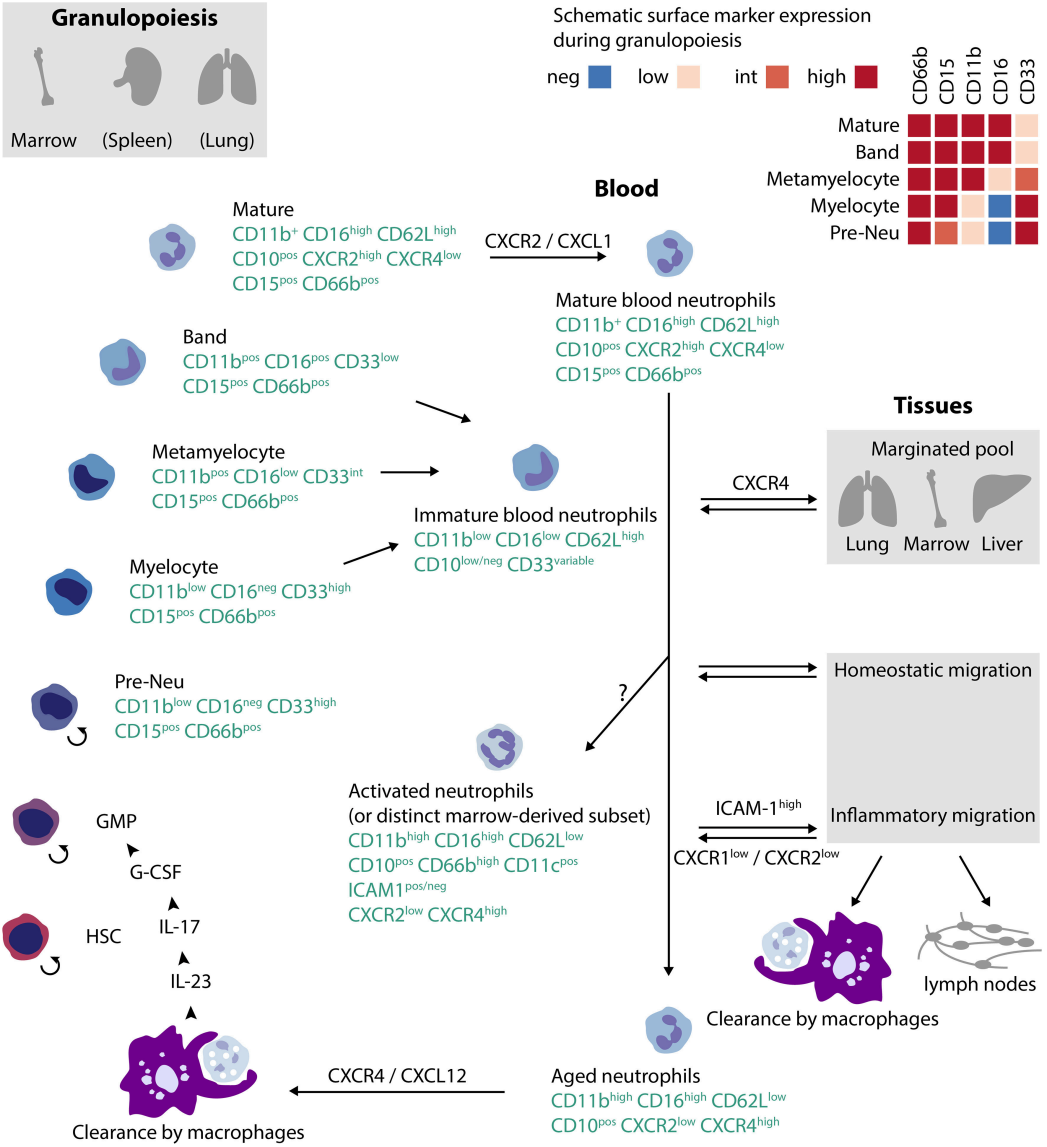
Lifecycle of human neutrophils. Neutrophils arise in bone marrow, spleen and (at least in mice) in lung from hematopoietic stem cells (HSC), progressing to committed granulocyte-monocyte progenitors (GMP), and then through a set of intermediate stages to mature neutrophils. Neutrophils exit to blood under the control of CXCR2, usually as mature cells but under conditions of stress also as immature cells. Over time, neutrophils age, expressing CXCR4 that mediates return to marrow. Alternate pathways for blood neutrophils include intravascular activation, intravascular margination, homeostatic migration into tissues, or migration into inflamed tissues. Clearance occurs via macrophages either in tissues or in bone marrow. The localization of the recently-defined preNeu in the previously-accepted neutrophil ontology (GMP → myeloblast → promyelocyte → myelocyte) remains uncertain; one plausible configuration is shown. The small circular arrow ↺reflects replication competence. References:(18–23).

Neutrophils arise from hematopoietic stem cells (HSCs) in bone marrow, spleen, and probably lung (Figure 1) (24, 25) HSCs give rise to multipotent progenitors (MPP), which yield common myeloid progenitors (CMP) and then granulocyte monocyte progenitors (GMP). The latter commit to a program to become monocyte/dendritic cells, mast cells, basophils, or neutrophil/monocytes (26). A proliferation-competent committed progenitor termed a preNeu develops into post-mitotic immature neutrophils (myelocytes, metamyelocytes, band cells) and finally segmented mature neutrophils (18). Immature neutrophils are also be found in peripheral blood in time of immunologic stress. Granulopoiesis is stimulated predominantly through the IL-23/IL-17/G-CSF axis and to a lesser extent by GM-CSF and M-CSF, although mice lacking all three colony stimulating factors still have ~10% of normal circulating neutrophils (19, 27). Other cytokines have also been implicated, for example IL-6, which has a special importance in emergency granulopoiesis in response to systemic infection (24, 28).

Studies in mice suggested a circulating neutrophil half-life of 1.5 h by exogenous labeling followed by transfer and 8–10 h after *in vivo* labeling (29, 30). In humans, endogenous labeling raised the possibility that the neutrophil lifespan may be as long as 5.4 days (half-life 3.7 days) (20). This surprising result reflects assumptions about the relationship between marrow and circulation that have been disputed, and more recent studies suggest instead a half-life of 19 h, conforming more closely to murine data and to conventional expectations (31, 32). *In vitro*, human neutrophils typically undergo apoptosis within 24 h, but >90% viability even after 9 days can be achieved in the presence of GM-CSF (33). Thus, whatever the basal half-life of circulating neutrophils, it is likely that some neutrophils live for a prolonged period *in vivo*, especially in an inflamed context.

Neutrophils released into circulation can follow several paths (Figure 1) (34). The simplest is uneventful aging followed by return to the bone marrow, a process influenced by the microbiome and mediated through progressive expression of the SDF-1 (CXCL12) receptor CXCR4 in older neutrophils (35–38). Production of mediators by marrow macrophages phagocytizing aged neutrophils in turn regulates granulopoiesis, forming a “neutrostat” that contributes to circadian release of fresh neutrophils into blood (36). Some circulating neutrophils marginate in lung, also under the influence of CXCR4, where they can combat infection locally or be released at need into the circulation (34, 39). A marginated neutrophil population is also observed in liver and bone marrow (40).

Neutrophils recruited to inflamed tissues undergo several distinct fates. Some die locally through apoptosis or other forms of cell death, including ejection of their DNA as neutrophil extracellular traps (NETs) (41). Neutrophils can egress from tissues via lymphatics, appearing in lymph nodes loaded with antigen (42). Neutrophils from inflammatory infiltrates can return to the circulation, a possibility originally visualized in zebrafish and subsequently observed in mice (38, 43, 44). More recently, it has been recognized that neutrophils enter some tissues even without an exogenous trigger. For example, neutrophils promote angiogenesis in the regenerating uterine lining and modulate metabolism in adipose tissue, particularly in obesity (34). In healthy mice, neutrophils have also been observed in liver, skin, intestine, skeletal muscle, kidneys, and heart, although not in brain or gonads (45). While the role of these patrolling neutrophils remains to be established, one function may be to modulate the activity of local macrophages by which they are phagocytosed at the end of their lifespan (45, 46).

## Neutrophil Heterogeneity as a Function of Maturity

The neutrophil lifecycle accounts for substantial phenotypic heterogeneity (Figure 1). Mature human neutrophils exhibit a characteristic multi-lobular nucleus and high surface expression of CD16 (FcγRIII), CD62L (L-selectin), and CD10 (neutral endopeptidase), along with neutrophil lineage markers CD15 and CD66b (21, 22). By contrast, immature neutrophils released from marrow after immune stress are CD16^lo^ and CD10^lo^, often but not invariably together with band nuclear morphology (22, 47, 48) Neutrophils less mature than band cells exhibit elevated CD33 (Siglec-3) and lower CXCR2, the receptor that enables mobilization out of the bone marrow niche (18, 21, 49). Immature neutrophils express more CXCR4 than mature neutrophils, likely promoting their retention in the bone marrow (18). As neutrophils age, expression of CXCR4 again increases, licensing return to the bone marrow for clearance (35). Aging is accompanied by other changes, including elevated expression of the integrins CD11b and CD11c, lower CD62L, and lower CD47, an inhibitor of phagocytosis (23). This maturation-related variation corresponds to changes in effector function. Aged neutrophils can be particularly effective in migration to sites of inflammation for immune defense, although immature neutrophils display superior bactericidal function against certain pathogens (50, 51). Murine spleen contains a mature Ly6G^hi^ population of motile, highly phagocytic neutrophils as well as immature Ly6G^lo^ neutrophils with a preserved capacity for mitosis, limited mobility, and low phagocytic capacity (49). In humans receiving G-CSF, immature CD10^neg^ neutrophils stimulate T cells while mature CD10^pos^ neutrophils are suppressive (22).

## Neutrophil Heterogeneity as a Function of Activation State

Beyond maturational changes, neutrophils shift phenotype with activation. Mobilization of intracellular granules brings not only soluble mediators but also pre-formed membrane proteins to the neutrophil surface. These include CD66b, the α and β chains of the β2 integrin CD11b/CD18 (Mac-1), and in some individuals CD177 with its associated protease proteinase 3 (PR3) (21, 52, 53). Other surface markers are lost, including CD62L, which is rapidly shed in activated neutrophils. Activated neutrophils exhibit multiple changes in function compared with resting neutrophils. Inside-out signaling and clustering enhance integrin binding, and activated neutrophils thus exhibit enhanced mobility as well as production of lipid mediators, cytokines, chemokines, and reactive oxygen species (54).

Neutrophils acquire additional surface markers that reflect their migratory history (Figure 1). Many neutrophils that transmigrate into inflamed tissue do not die by apoptosis, as was previously assumed, but rather migrate back into the circulation (38, 43, 44). In human neutrophils, *in vitro* reverse transendothelial migration correlates with the appearance of surface ICAM-1 (CD54), elevation of CD18, and lower CD62L, CXCR1 and CXCR2 (55). In mice, reverse-migrated neutrophils are characterized by ICAM-1 and upregulation of CXCR4 through which they can “transplant” inflammation from the periphery to the lung before returning to the bone marrow for final clearance (38, 44, 56). ICAM-1 elevation has also been reported in human neutrophils after prolonged *in vitro* stimulation and in a CD16^hi^CD62L^lo^CD11b^hi^CD11c^hi^ peripheral blood neutrophil population induced in normal donors treated with i.v. LPS, and hence is not restricted to reverse-migrated neutrophils (20). Thus healthy donors exhibit an almost uniform signature of mature neutrophils in blood, CD11b+CD16^hi^CD62L^hi^CD10^hi^. After administration of LPS or G-CSF, additional populations appear, including immature cells (CD11b^lo^CD16^lo^CD62L^hi^CD10^lo^, banded nuclear morphology) and cells with a phenotype suggestive of activated mature cells (CD11b^hi^CD16^hi^CD62L^lo^CD10+, increased nuclear lobulation) (20, 22) Intriguingly, this latter subgroup can be found even in normal marrow; labeling studies suggest a similar age to mature neutrophils, raising the possibility that some CD62L^lo^ cells may not be mature neutrophils activated intravascularly but rather a distinct type of neutrophil released directly from marrow under stress (57).

## Neutrophil Heterogeneity Beyond Aging and Activation

The broad phenotypic variability associated with maturation and activation complicates the task of discerning additional axes of heterogeneity in the form of discrete neutrophil subsets. This topic has been expertly reviewed (40, 58, 59). We will focus on targetable neutrophil heterogeneity by limiting consideration here to three areas: low-density neutrophils, immunomodulatory neutrophils, and neutrophil subgroups defined by the surface marker CD177.

### Low-Density Neutrophils

The average density of neutrophils from healthy subjects is >1.080 g/ml and therefore higher than lymphocytes (1.073–1.077 g/ml) and monocytes (1.067–1.077 g/ml) (60). Density gradient centrifugation leverages these differences to separate PBMC from granulocytes. Low-density neutrophils (LDN, also termed low-density granulocytes) are neutrophils found in the PBMC layer rather than the granulocyte pellet (61, 62). On microscopic examination, many display an immature nuclear morphology, and gene expression studies suggest immaturity of the population as a whole, although expression of CD10 and other markers of maturity (e.g., CD16^hi^) suggest that not all LDN are neutrophils released prematurely from the marrow (22, 62, 63). Importantly, normal-density neutrophils (NDN) exposed to sera containing complement or immune complexes can also segregate with PBMC, highlighting the dynamic nature of density as a physical property of neutrophils that reflects factors such as granule content and cytoplasmic volume (22, 61). Elevated CD66b and CD11b further suggest that some LDN represent activated mature cells (62). In mice, interconversion between LDN and NDN occurs in neutrophils adoptively transferred into live animals, while *ex vivo* TGF-β treatment induces LDN-like features in NDN (64). Thus, LDN likely represent a diverse population of immature and activated mature neutrophils.

Elevation in peripheral blood LDN has been observed in many states of immune stress, including acute rheumatic fever, JIA, RA, systemic lupus erythematosus (SLE), autoinflammatory diseases, G-CSF administration, cancer, and sepsis (61–67). Their characteristics vary widely with context, and can for example include enhanced production of pro-inflammatory cytokines such as TNF and type I interferons, spontaneous NET generation (discussed further below), and immunosuppressive capacity (40). LDN thus reflect the phenotypic and functional plasticity of the neutrophil lineage. Whether some LDN also represent a discrete, stable neutrophil subset remains to be determined.

### Immunomodulatory Neutrophils

The view of neutrophils as simple foot-soldiers of immunity has given way to a more nuanced understanding of these cells as full participants in the immune network. Examples of the reciprocal interchange between neutrophils and adaptive immunity are abundant. Neutrophils home to lymph nodes in response to CCL19 and CCL21, carrying antigen for presentation to T cells in the context of MHC II and the canonical costimulatory molecules CD80 and CD86 (68–74). Neutrophils can differentiate into cells with surface and functional similarity to dendritic cells (75, 76). CD15^int/lo^CD16^int/low^CD11b^high^ “B helper” (N_BH_) neutrophils have been reported in the marginal zone between lymphoid white pulp and non-lymphoid red pulp of human spleen than interact with B cells to promote IgM production and Ig class switching (77). The N_BH_ phenotype develops under the influence of IL-10 from local cells, including splenic endothelial cells, and confers the capacity to produce mediators including APRIL, BAFF, and IL-21. Of note, not all investigators have observed these cells in human spleen, such that further exploration of N_BH_ cells is required (78).

Neutrophils can also suppress adaptive immunity. This capacity has gained particular attention in cancer biology, where neutrophils can promote tumor growth by inhibiting responding lymphocytes (64, 79, 80). Mechanisms include arginase-1 to deplete extracellular arginine required for T cell function, reactive oxygen and nitrogen species to impair effector T cells in favor of regulatory T cells, IL-10, and TGF-β as immunosuppressive mediators, and pathways mediated through direct cell-cell contact (20, 34, 81). Myeloid cells with the capacity to block T cell activation (and under some conditions B cells and NK cells) have been termed myeloid-derived suppressor cells (MDSC), a loosely-defined category now recognized to include both neutrophil-like and monocyte-like cells (82, 83). Neutrophilic MDSC (so-called PMN-MDSC) are typically considered relatively immature, but in G-CSF-treated donors suppressive capacity in fact resides within the mature (CD10+) fraction, both LDN and NDN (22, 83). Suppressive capacity can also be elicited *ex vivo* in healthy-donor neutrophils exposed to TLR ligands, consistent with *in vivo* LPS challenge data (20, 81). Immunosuppressive capacity is thus available to neutrophils at a range of maturational states with appropriate stimulation. It remains to be established whether this capacity represents part of a broader differentiation program in a limited group of neutrophils—i.e., whether PMN-MDSC represent one or more distinct neutrophil subsets.

### CD177

Another protein expressed dichotomously in human neutrophils is CD177, originally known as NB1 (84). A glycoprotein of ~60 kD attached to the neutrophil surface via a GPI linker, CD177 is present on 40–60% of neutrophils in most donors, with a range extending from 0 to 100%; some individuals manifest a CD177^int^ population as well (52, 85, 86). Like another dichotomously-expressed neutrophil protein olfactomedin 4 (OLFM4), CD177 is localized to the specific granules, residing in the granule membrane for rapid mobilization to the surface with cell activation; however, CD177^pos^ and OLFM4^hi^ subsets otherwise exhibit no interdependence (52, 87).

The function of CD177 is incompletely understood. Lacking a transmembrane domain, CD177 cannot itself transmit a signal intracellularly, but antibody ligation studies show that CD177 can signal through the β2 integrins with which it associates in *cis* at the neutrophil surface (88, 89). Resulting enhancement in integrin expression and affinity translate CD177 ligation into neutrophil arrest, blocking transmigration (89). CD177 thus functionally echoes murine Ly6G, a neutrophil-restricted GPI-linked protein from the same Ly6/UPAR protein family that also interacts with β2 integrins and can modulate neutrophil migration, although Ly6G ligation appears to impair rather than enhance integrin binding (15, 90, 91). The endogenous receptor for CD177 remains uncertain. *In vitro* data implicate the endothelial adhesion molecule PECAM-1; however CD177^pos^ neutrophils display no particular affinity for PECAM-1-expressing platelets or *in vivo* migratory advantage, rendering the physiological significance of the *in vitro* observations uncertain (89, 92–94) Interestingly, CD177 specifically binds the neutrophil protease PR3, which is stored primarily in azurophilic and specific granules in resting neutrophils and mobilized to the membrane during activation, such that CD177^pos^ cells are identical to PR3^pos^ cells among activated neutrophils (95–97) Some data suggest that PR3 may promote the migration of CD177^pos^ neutrophils, but more recent data indicate that CD177 binding impairs PR3 function, leaving the functional implications of the CD177-PR3 interaction uncertain (92, 98).

The basis for the expression of CD177 in some neutrophils but not others is partially understood. *CD177* resides adjacent to a related pseudogene *CD177P1* that is characterized by a stop codon in the region corresponding to *CD177* exon 7. Through a process of homologous recombination (gene conversion), approximately 12% of *CD177* alleles feature the *CD177P1* stop codon and thus represent null variants. Accordingly, the observed allelic distribution matches that expected by Hardy-Weinberg equilibrium, with 78% WT/WT, 19% WT/null, and 3% null/null (85, 99). In subjects with 2 intact copies of *CD177*, the CD177^pos^ fraction is typically 50–98%; in WT/null, 10–60%; and in null/null 0%. Why some neutrophils from WT/WT donors lack CD177 expression remains undefined, but presumably reflects epigenetic regulation (100). Interestingly, in individuals with 2 intact copies of *CD177*, epigenetic control enforces expression of single parental allele in all CD177-expressing neutrophils, although the purpose of such tight control is unknown (101).

Distinct immunological roles of CD177^pos^ and CD177^neg^ neutrophils have not yet been established. Individuals with nearly 100% CD177^pos^ neutrophils or lacking CD177 altogether appear healthy. The proportion of neutrophils expressing CD177 in an individual typically remains stable over time but rises in pregnancy, sepsis, and pathologic conditions including polycythemia vera, vasculitis, and SLE (86, 102). Studies of circulating CD177^pos^ and CD177^neg^ neutrophils reveal similar expression of integrins and Fc receptors, fibronectin adhesion, *in vitro* migration, and reactive oxygen species production (89, 103). Gene expression profiling using microarrays identified minor differences, principally in genes encoding granule proteins, although protein levels remained similar (104). Surface PR3 has been proposed as a potential modulator of T cell proliferation (105). A recent study suggested that CD177^pos^ cells express enhanced bactericidal capacity as well as IL-22 production, reflecting a potentially protective role in inflammatory bowel disease, including through use of a murine model (106). However, the use of an antibody clone that is known to activate neutrophils (MEM-166) to sort CD177 populations complicate the interpretation of these data, as functional measures may become confounded by the activation-mediated effect of the antibody itself (89). CD177 may be of interest in vasculitis, as it can mediate the tethering of PR3, the target of c-ANCA autoantibodies to the neutrophil surface.

## Migratory Pathways as a Wedge Between Protective and Pathologic Functions in Neutrophils

Beyond population heterogeneity, opportunities for intervention in neutrophil biology could emerge through effector pathways that are employed differentially in pathogenic and defensive functions. Given the myriad effector pathways employed by neutrophils, it is likely that there are multiple such opportunities. For example, in zebrafish, H_2_O_2_ is required for initiation of neutrophil recruitment to wounding but dispensable for migration toward injected bacteria (107). The zebrafish IL-1β ortholog and its downstream signaling partner MyD88 are similarly required for neutrophil recruitment triggered by wounding but not bacteria (108). We will focus here on another intriguing discrepancy between sterile and septic neutrophil migration related to the role of neutrophil β2 integrins.

The leukocyte recruitment cascade is well-established (54, 109). Circulating neutrophils roll across the endothelial surface under the influence of adhesive interactions between endothelial P- and E-selectins and neutrophil ligands including PSGL-1, further slowed by weak, transient interactions between other receptor-ligand pairs such as endothelial ICAM-1 and low-affinity neutrophil β2 integrins. With activation, endothelial cells upregulate these adhesion molecules and neutrophils augment the quantity and affinity of surface integrins, resulting in neutrophil arrest. Further neutrophil activation via chemokines presented on the endothelial glycocalyx and/or transported by endothelial cells to the luminal surface solidifies the attachment through post-adhesion strengthening (54, 110). Adherent neutrophils crawl in an integrin-dependent manner to sites suitable for transmigration between or through endothelial cells and then along sub-endothelial pericytes to sites of eventual egress into tissue (111, 112). While much of this cascade has been defined in mice, human relevance is supported by the susceptibility to infection in patients lacking the β2 chain CD18 (113).

Yet this selectin-integrin paradigm is not the whole story. Neutrophils lacking all integrins can migrate through the 3-dimensional matrix of tissue interstitium via amoeboid motion (“flowing and squeezing”) (114) Mice lacking β2 integrins or subject to integrin blockade still mount neutrophilic infiltrates. In particular, neutrophils can enter the airway without β2 integrins, although entry into the pulmonary parenchyma exhibits partial integrin dependence, as shown in studies employing adoptive transfer of mixed wild-type and CD18–/– neutrophils (90, 115–120) Indeed, under certain circumstances integrins slow neutrophil migration into lung, such that impairing integrins actually promotes neutrophil entry (116, 120). Consistent with these murine findings, neutrophilic pneumonia is observed in humans and cows lacking CD18, although recurrent pulmonary infections remain a clinical feature of this immunodeficiency in both species (113, 121, 122). In peritoneum, migratory impairment resulting from integrin deficiency or blockade is partial, with marked variability among experimental systems (90, 115, 116, 123–125). In the liver, integrins mediate neutrophil accumulation after thermal injury but are dispensable for migration induced by live bacteria in favor of CD44-hyaluronan adhesion (126). In other sites, β2 integrins play a more clear-cut role, including skin and in joints inflamed through immune complex deposition, although at least in joints the initial dependence on integrins may become less prominent as inflammation proceeds (13, 110, 114, 116, 127–129). Thus, mechanisms employed by neutrophils to enter tissues vary with site and also with stimulus, via pathways not limited to the classic leukocyte adhesion cascade.

Could this variability be exploited therapeutically? In liver, sterile infiltration is dependent on β2 integrins, while these integrins appear dispensable for septic infiltration (126). The generalizability of this principle was tested in murine peritoneum. Co-transfer of WT and CD18–/– neutrophils identified a markedly greater role for β2 integrins in sterile than septic peritonitis (i.p. IL-1β vs. live *E. coli*) (90). Correspondingly, targeting integrin-mediated neutrophil recruitment via an antibody directed against the integrin modulator Ly6G attenuated only sterile neutrophil infiltration, and then only in neutrophils expressing CD18. Consistent with the known variation in integrin dependence, ligation of Ly6G attenuated integrin-dependent arthritis and integrin-mediated post-adhesion strengthening on inflamed cremaster muscle but had no effect on integrin-independent neutrophil infiltration into lung (15, 90). These findings suggest that neutrophil migration in sterile disease could potentially be targeted without impairing antimicrobial defense. To the extent that integrin compromise can be rendered neutrophil-selective, as with Ly6G ligation, blockade is unlikely to phenocopy human CD18 deficiency, which impacts not only neutrophils but also monocytes, macrophages, and T cells. Humans do not express *Ly6G*, but *CD177* could potentially fulfill a similar role, given its similar structure, selective expression in neutrophils, spatial association with β2 integrins, and capacity to block neutrophil migration upon ligation (88, 89, 91, 93). Anti-CD177 (clone MEM166) arrests migration by enhancing integrin-mediated adhesion via mechanisms including inside-out signaling and impaired integrin recycling (89). Since the relevant endogenous counterligands of both Ly6G and CD177 are unknown, it remains unclear if this difference in effect reflects intrinsic differences between these proteins or variability among the available targeting antibodies.

Importantly, not only integrin binding but also timely integrin release is required for successful transmigration. Interference with this step through interventions that prevent integrin affinity modulation represent a further opportunity for intervention in migration, a “leukadherin”-type mechanism (120, 130). To date, however, this approach lacks specificity for neutrophils.

## Neutrophil Targeting in Inflammatory Diseases

Heterogeneity within the neutrophil population and in the pathways that neutrophils employ to access inflamed tissues represent opportunities for intervention in neutrophil-mediated disease. None of these have yet been explored definitively, so this discussion remains necessarily speculative. We will focus on three diseases: inflammatory arthritis, SLE, and vasculitis.

### Inflammatory Arthritis

This disease family encompasses conditions including JIA, adult RA, and crystalline arthropathies such as gout (131). The presence of neutrophils in the joint fluid is the *sine qua non* of active inflammation across this spectrum. Experimental data across species implicate neutrophils in both initiation and perpetuation of disease (13–15, 132–134). Neutrophils stimulated via C5a arrest at the synovial endothelium in a β2 integrin-dependent manner and transmigrate under the influence of leukotriene B_4_ (LTB_4_) and other chemokines (110, 135, 136). Within the joint, activated neutrophils provide LTB_4_ and IL-1β that amplify the inflammatory process (13, 136, 137). Abundant in synovial fluid, neutrophils remain sparse in synovial tissues, although they can be observed in the inflamed pannus early in disease and at the cartilage-pannus junction (138, 139). Their proteases can injure cartilage, including through “frustrated phagocytosis” of embedded immune complexes (140). More recently, neutrophils have been recognized as a source of citrullinated autoantigens in seropositive RA at sites including joint, oropharynx and lung (141–147). Interestingly, not all neutrophil activity in arthritis is pathogenic. Neutrophil microvesicles can protect cartilage by promoting local production of TGF-β (148). In gout, aggregated NETs can help resolve flares through protease-mediated clearance of pro-inflammatory mediators, though the practical contribution of these mechanisms remains unclear (149, 150).

Neutrophils likely play roles in arthritis beyond their immediate impact within the joint environment. Their capacity for regulation of B cells and T cells, and for transport and presentation of antigen, has been noted above. Neutrophils provide mediators that contribute to systemic inflammation. For example, they are a major source of the pro-inflammatory calgranulins S100A8/A9 and S100A12, danger-associated molecular pattern (DAMP) proteins that can activate other cells via pathways including TLR4 ligation (151–153). In systemic JIA, a form of childhood arthritis characterized by fever and rash, concentrations of these mediators in blood are highly elevated, correlating with circulating neutrophil counts and potentially contributing to IL-1β release by monocytes and other cells (48, 154). Recent studies have identified a specific expansion of hypersegmented CD16^pos^ CD62L^dim^ neutrophils in patients with systemic JIA with active, systemic symptoms compared to patients with active arthritis or inactive disease (155). These changes in phenotype and count are accompanied by a sepsis-like transcriptomic pattern in systemic JIA circulating neutrophils (67).

Points of intervention in neutrophil biology in arthritis range across a broad spectrum, including recruitment, effector pathways, and antigen generation. Mechanisms of recruitment blockade in mice include neutrophil-specific integrin blockade and chemokine antagonism (15, 90, 156). Targeting toxins and other compounds to neutrophils, e.g., via scavenger receptors, could potentially hasten resolution of inflammation (157). A similar strategy could alter the ability of neutrophils to generate citrullinated autoantigens, for example by introducing inhibitors of peptidylarginine deiminase enzymes (158). Of note, RA was one of the first diseases to be associated with LDN (61). Induction of the LDN phenotype by RA plasma, complement, or aggregated IgG suggests that LDN could represent an activated and/or degranulated cell population (61, 159). Direct *ex vivo* analysis of RA LDN has found markers of immaturity but failed to identify the enhanced capacity for NET formation observed in SLE LDN, such that their role in antigen generation remains uncertain (63).

### Systemic Lupus Erythematosus

The evidence for a role for neutrophils in SLE is now compelling, as has been reviewed in depth (158, 160, 161) Most of the attention has focused on LDN and NETs. The presence of neutrophils in PBMC preparations was described originally in a cohort of diseases including SLE, and it was in SLE that the term “low density granulocytes” was first applied (61, 62). LDN within SLE have several features that suggest a role in disease pathogenesis. They can elaborate type I interferons and generate NETs *in vitro* without exogenous stimuli, expose SLE-associated autoantigens and stimulate interferon production by plasmacytoid dendritic cells. (62, 162–164). Free DNA has been observed in lupus nephritis kidneys, potentially reflecting an impaired ability to clear NETs (165). Indeed, some SLE patients exhibit autoantibodies against DNase I, the enzyme primarily responsible for NET clearance, or against the NET themselves that block enzymatic attack (165). NETs can be elicited by anti-phospholipid antibodies, potentially contributing to elevated thrombosis risk in SLE (166). Finally, neutrophils may contribute to aberrant B cell development in SLE bone marrow through production of IFNα and B cell growth factors (167).

This substantial evidence base renders neutrophils, and particular NETosis, an intriguing therapeutic target in SLE (158). Interference with NETosis can ameliorate manifestations of experimental SLE (168, 169). Importantly, however, genetic deficiency of PAD4 and other pathways required for NETosis can have no effect or even worsen SLE-like disease in mice (170–172). Which murine studies replicate the human potential of NET blockade remains to be determined. Depleting or blocking LDN could represent an avenue forward, but will require further understanding of their origin and role in immune defense as well as in pathogenic inflammation.

### Vasculitis

Inflammatory disease of blood vessels can assume many forms, with a severity ranging from trivial to catastrophic. Neutrophilic infiltration into blood vessels is a common feature of vasculitis. For example, neutrophils are the dominant tissue leukocyte in immune complex-mediated leukocytoclastic vasculitis and in inflamed coronary arteries in Kawasaki disease (173–175). Neutrophils may mediate Henoch-Schönlein purpura (HSP), the most common childhood vasculitis, through their ability to recognize the Fc portion of IgA molecules (176). In anti-neutrophil cytoplasmic antibody (ANCA)-associated vasculitis and related *in vivo* disease models, the neutrophil enzymes PR3, and myeloperoxidase are targeted by autoantibodies and play a potential pathogenic role, mediating neutrophil activation and β2 integrin-dependent adhesion to endothelium (177–182). For PR3, the subset of neutrophils expressing CD177 may play a particularly important role, because CD177 binds PR3 to enable surface expression at high level (92, 96). This binding enables anti-PR3 antibodies to activate neutrophils by signaling via CD177-associated integrins, potentially similar to the activation of neutrophils via anti-CD177 antibodies (88, 89, 103). Of note, PR3 may be expressed via CD177-independent pathways, and CD177 expression is not an invariable requirement for neutrophil-mediated inflammation induced via anti-PR3 antibodies (102, 183). ANCAs bound to the neutrophil surface also activate neutrophils via their surface Fc receptors and can trigger NETs that contribute to tissue injury (184–186). Detailed mechanistic understanding of the role of neutrophils in non-ANCA vasculitis remains more limited, but it is plausible to suspect that their presence in these sterile inflammatory infiltrates reflects a pathogenic role (187).

These considerations render neutrophils an interesting target population in vasculitis. In some patients, there is an emergent need to shut down inflammation to protect affected tissues including lung, kidney, brain, nerve, and heart. In such cases, short-term infectious risk might be a tolerable exchange for rapid cessation of disease activity. In other cases, vasculitis is chronic and indolent, and a more careful balancing act is required. The neutrophil populations and pathways to be targeted vary with the disease. These include CD177^pos^ neutrophils in anti-PR3 ANCA-associated vasculitis, which could be depleted or treated to interfere with the ability of CD177 to bind PR3, although non-CD177-mediated PR3 expression may limit the effectiveness of such a strategy (102). Blockade of neutrophil β2 integrins could attenuate vasculitis mediated through firm adhesion between neutrophil and endothelium. Intracellular activation pathways and NETs have also been proposed as targets (188, 189).

## Conclusions

Neutrophils exhibit a broad range of phenotypes. Much of this variability reflects developmental stage and activation status, integrating both stimulatory exposures and migratory history. As a result, neutrophils diverge from one another in nuclear morphology, buoyancy, surface markers, migratory, and phagocytic capacity, NET generation, and immunomodulatory function, among other characteristics. There remains intense interest in the possibility that this diversity manifests specific developmental programs to which individual neutrophils become committed, reflecting thereby true neutrophil subsets. However, to date evidence in favor of discrete subsets is insufficient to reject the alternative hypothesis that phenotypic variation reflects the impact of diverse environments on neutrophils within a single developmental continuum. The growing capacity for single-cell analysis of immune populations will likely provide important insights into this biology in coming years.

For the purposes of therapeutic targeting, the ontogeny of neutrophils is less important than the fact of their phenotypic diversity, now well-established if still incompletely delineated. This diversity opens the possibility of targeting neutrophils engaged in disease pathogenesis without similarly perturbing neutrophils engaged in antimicrobial defense. Such “wedge opportunities” arise not only with respect to heterogeneity within neutrophil population but also, somewhat less appreciated, in the pathways employed by neutrophils to respond to different stimuli. We reviewed here the evidence in favor of a greater role for neutrophil β2 integrins in neutrophil migration toward sterile than septic triggers, at least in some sites, and the potential role for neutrophil-specific integrin modulators (Ly6G in mice, potentially CD177 in humans) to enable lineage-specific integrin targeting. Better understanding of neutrophil biology will open further possibilities for the selective manipulation of this lineage in human therapeutics.

## Author Contributions

RG-B and PAN conceptualized and wrote the review and created the figure.

### Conflict of Interest Statement

The authors declare that the research was conducted in the absence of any commercial or financial relationships that could be construed as a potential conflict of interest.
